# Resilience in Alzheimer's disease: Impact of operationalization and methodological choices

**DOI:** 10.1002/alz.70185

**Published:** 2025-04-28

**Authors:** Sophie Mutel, Lara Quatrocchi, Daniele Altomare, Christian Chicherio, Max Scheffler, Karl‐Olof Lövblad, Kaj Blennow, Nicholas J. Ashton, Henrik Zetterberg, Marc Abramowicz, Jean‐Louis Blouin, Chen Wang, Carine Wyss‐Dominguez, Augusto J. Mendes, Valentina Garibotto, Giovanni Frisoni, Federica Ribaldi

**Affiliations:** ^1^ Laboratory of Neuroimaging of Aging (LANVIE) University of Geneva Geneva Switzerland; ^2^ Geneva Memory Centre Department of Rehabilitation and Geriatrics Geneva University Hospitals Geneva Switzerland; ^3^ Competence Centre on Ageing (CCA) Department of Business Economics Health and Social Care (DEASS) University of Applied Sciences and Arts of Southern Switzerland (SUPSI) Manno Switzerland; ^4^ Center for Interdisciplinary Study of Gerontology and Vulnerability (CIGEV) University of Geneva Carouge Switzerland; ^5^ Division of Radiology Geneva University Hospitals Thonex Switzerland; ^6^ Neurodiagnostic and Neurointerventional Division Diagnostic Department Geneva University Hospitals Geneva Switzerland; ^7^ Department of Psychiatry and Neurochemistry Institute of Neuroscience and Physiology The Sahlgrenska Academy at the University of Gothenburg Göteborg Sweden; ^8^ Clinical Neurochemistry Laboratory Sahlgrenska University Hospital Mölndal Sweden; ^9^ Paris Brain Institute ICM Pitié Salpêtrière Hospital Sorbonne University Paris France; ^10^ Neurodegenerative Disorder Research Centre Division of Life Sciences and Medicine and Department of Neurology Institute on Aging and Brain Disorders University of Science and Technology of China and First Affiliated Hospital of USTC Hefei China; ^11^ Centre for Age‐Related Medicine Stavanger University Hospital Stavanger Norway; ^12^ King's College London Institute of Psychiatry Psychology & Neuroscience Maurice Wohl Clinical Neuroscience Institute London UK; ^13^ NIHR Biomedical Research Centre for Mental Health & Biomedical Research Unit for Dementia at South London & Maudsley NHS Foundation London UK; ^14^ UK Dementia Research Institute at UCL London UK; ^15^ Department of Neurodegenerative Disease, UCL Institute of Neurology London UK; ^16^ Hong Kong Centre for Neurodegenerative Diseases Hong Kong Science Park, Shatin Hong Kong China; ^17^ Wisconsin Alzheimer's Disease Research Centre University of Wisconsin School of Medicine and Public Health, University of Wisconsin–Madison Madison Wisconsin USA; ^18^ Genetic Medicine Division University Hospitals and University of Geneva Geneva Switzerland; ^19^ Laboratory of Neuroimaging and Innovative Molecular Tracers (NIMTlab) Geneva University Neurocentre and Faculty of Medicine, University of Geneva Geneva Switzerland; ^20^ Division of Nuclear Medicine and Molecular Imaging Geneva University Hospitals Geneva Switzerland; ^21^ Centre for Biomedical Imaging University of Geneva Geneva Switzerland

**Keywords:** Alzheimer's disease resilience, brain resilience, cognitive decline, cognitive resilience, residual approach, residual correction methods, risk factors

## Abstract

**INTRODUCTION:**

Resilience, the ability to maintain cognition or brain integrity despite Alzheimer's disease (AD) pathology, is often quantified using the residual approach. However, the variability in methodology and correction methods for this approach raises concerns about the interpretability of findings across studies.

**METHODS:**

We assessed brain resilience (BR) and cognitive resilience (CR) in a memory clinic population using the residual approach. We compared non‐corrected and corrected residuals’ associations with risk factors using linear regression models, and their impact on longitudinal cognition using linear mixed‐effects models.

**RESULTS:**

Corrected versus non‐corrected BR yielded distinct, often opposing, associations. For example, glial fibrillary acidic protein (GFAP) was negatively associated with non‐corrected BR (β = –0.33; *p* < 0.01) but positively with corrected BR (β = 0.5, *p* < 0.001). Only corrected CR measures yielded significant associations. Only corrected residuals predicted cognitive decline.

**DISCUSSION:**

The observed discrepancies raise questions about the reliability of the residual approach in accurately capturing resilience.

**Highlights:**

- Corrected and non‐corrected residuals show distinct associations with risk factors.
- Corrected and non‐corrected residuals show different predictions of cognitive decline.
- These approaches may reflect general brain health rather than true resilience mechanisms.

## 1 BACKGROUND

Alzheimer's disease (AD) is characterized by an abnormal accumulation of amyloid beta plaques (A) and neurofibrillary tangles of hyperphosphorylated tau (T) in the brain. However, the impact of these neuropathological changes on brain structure and cognition varies widely among individuals.^1^ Resilience, defined as the maintenance of brain integrity (brain resilience [BR]) or cognitive function (cognitive resilience [CR]) despite neuropathology, could explain this variability.^2^, ^3^ Identifying factors underlying resilience mechanisms may aid in developing accurate prognostic measures and novel interventions for AD.

Currently, there is a lack of standardization in resilience assessment methods. Some studies rely on single proxies like education or occupation,^4^, ^5^ others use questionnaires like the Cognitive Reserve Index questionnaire (CRIq),^6^ assessing a broader range of factors. Resilience can also be defined as maintaining cognition scores above a defined threshold in the presence of AD‐related pathological brain changes.^7^, ^8^ This heterogeneity in methods has produced inconsistent findings, making it challenging to identify the true drivers of resilience. For instance, younger age has been associated with both lower^8^ and higher^9^ resilience. Even education, a commonly used proxy, has shown mixed results, with some studies demonstrating a positive association with resilience^7^, ^10^ while others report no significant effect.^11^, ^12^

A popular method to quantify resilience is the residual approach^13^, which defines resilience as the variance in brain or cognitive measures that remains unexplained after accounting for the observed level of neuropathology. Residuals from a regression model, with brain or cognitive measures as the dependent variables and pathology as the independent variable, are used as individual resilience scores. This approach offers continuous measures, enabling a more sophisticated analysis than categorical definitions based on thresholds.

However, residuals are inherently correlated with the dependent variable of the regression used to extract them, especially when the correlation between dependent and independent variables is weak. For example, with a correlation of 0.7 between the variables, 50% of the residual variance is still explained by the dependent variable.^14^ In clinical settings, correlations between brain health or cognitive measures and neuropathology measures are typically lower, resulting in residuals that are closely associated with the dependent variable. In research on brain aging, in which residuals are also used, correction methods are often used to address this issue, but they are less common in AD resilience research. Here, we briefly describe two correction methods frequently used in brain aging studies.^15^, ^16^, ^17^ In the first method, termed “residual correction” here, a secondary regression uses the original residuals as the outcome and the original dependent variable as a predictor. The resulting residuals from this secondary regression are then used as the corrected residuals. The second method, termed “covariate correction,” includes the original dependent variable as a covariate in any further analyses using the residuals as predictors. Both methods effectively remove the variance explained by the original dependent variable.

Elman et al.^14^ highlighted that these correction methods, while removing correlation with the original dependent variable, introduce a dependence on the independent variable. Rather than being correlated with the measure of brain or cognitive state, corrected residuals are correlated with the measure of neuropathology. This close association between residuals and the original measures raises concerns about the ability of the residual approach to truly capture resilience as a distinct construct. Despite these concerns, the residual approach remains widely used,^18^ with inconsistent correction practices. This lack of methodological standardization has serious repercussions, as correction can alter the observed relationships between resilience and clinical or demographic factors, and between resilience and cognitive decline.^14^

A lack of direct comparisons within memory clinic populations limits our understanding of the practical implications of the residual approach to resilience assessment in clinical and research settings. This study explicitly addresses this gap by assessing resilience using the residual approach within a memory clinic population with mild cognitive impairment (MCI), a crucial stage for observing and potentially intervening on resilience mechanisms. The study aims to:

1. Evaluate how BR and CR are associated with biomarkers and demographic and clinical factors when calculated using non‐corrected versus corrected (residual correction and covariate correction) residuals.
2. Evaluate the influence of residual‐based resilience on cognitive trajectories.
3. Evaluate how cognitive resilience is associated with demographic and clinical factors when calculated using another resilience assessment method, the CRIq, in a separate population.

**RESEARCH‐IN‐CONTEXT**

1. **Systematic review**: We conducted a literature search using traditional databases (e.g., PubMed) to identify studies on resilience in Alzheimer's disease, focusing on the residual approach and its methodological variations.
2. **Interpretation**: Our study provides direct evidence that methodological choices within the residual approach significantly influence the identification of factors associated with resilience, as well as residuals’ prediction of cognitive decline. The results highlight the need for caution when interpreting results from cross‐sectional resilience assessments methods, which may primarily reflect general aspects of brain health rather than true resilience mechanisms.
3. **Future directions**: Future research should prioritize the development of new resilience assessment methods that incorporate multiple factors and longitudinal data. A multifaceted approach will enable a more comprehensive understanding of resilience and facilitate the study of associated factors and the influence on cognitive decline.

## 2 METHODS

### 2.1 Participants

This study included 245 participants from the memory clinic of Geneva University Hospitals (Geneva, Switzerland) who underwent various clinical, neuropsychological, and neuroimaging assessments according to clinical needs and, for some, as part of other ongoing research projects. The characteristics of the participants are presented in Table 1. Data collection was from 2014 to 2023. General inclusion criteria were the following: (1) age > 40 years, (2) no major psychiatric disorder, (3) no contraindications to magnetic resonance imaging (MRI), (4) no severe behavioral disturbance, (5) no severe disease or other life‐threatening condition within the last 5 years, and (6) no severe systemic disease. See Ribaldi et al.^19^ for further details on participant selection.

**TABLE 1:** Demographics and clinical characteristics of the two study groups (MCI and CN).

Variable	MCI (*n* = 121)	CN (*n* = 124)	*p*‐value
Age, years	72.4 (7.4)	61.7 (8.7)	**<0.001**
Education, years	13.5 (3.7)	15.5 (3.5)	**<0.001**
Sex, female	55 (45)	90 (73)	**<0.001**
Smoker	9 (7)	10 (8)	0.939
Hypertension	48 (40)	14 (11)	**<0.001**
Depression	29 (24)	30 (24)	0.493
Cardiovascular disease	20 (17)	13 (10)	0.608
Hypercholesterolemia	50 (41)	18 (15)	**<0.001**
MMSE score	26.5 (2.5)	28.8 (1.1)	**<0.001**
Hippocampal vol., mm^3^	7055 (960)	8283 (1031)	**<0.001**
Amyloid centiloid	49.8 (49.1)	4.9 (15.6)	**<0.001**
Tau SUVr	1.3 (0.3)	NA	NA
WMH, mm^3^	4843 (8659.3)	NA	NA
GFAP plasma levels	191.9 (109.4)	NA	NA
NfL plasma levels	23 (11.3)	NA	NA
*APOE* ε4 carrier	31 (26)	34 (27)	0.212

*Note*: Values represent means (SD) for continuous variables and *n* (%) for categorical variables. Mann‐Whitney tests were used to assess differences between groups for continuous variables. Chi‐squared tests of independence were used to assess differences between groups for categorical variables.

Abbreviations: *APOE*, apolipoprotein E; CN, cognitively normal; GFAP, glial fibrillary acidic protein; MCI, mild cognitive impairment; MMSE, mini‐mental state examination; NfL, neurofilament light chain; SUVR, standardized uptake value ratio; WMH, white matter hyperintensities.

The first population (*n* = 121) consisted of participants with MCI. MCI was defined based on the following clinical criteria: (1) objective evidence of cognitive impairment, (2) cognitive concern reported by the patient and/or informant (family or close friend), and (3) no functional impairment in activities of daily life. Participants in this population underwent clinical and neuropsychological assessment, MRI, amyloid positron emission tomography (PET), tau PET, and blood collection.

A separate population (*n* = 124) consisted of cognitively normal (CN) participants (volunteers, individuals with subjective cognitive decline, and worried well subjects) who also underwent clinical and neuropsychological assessment (including the CRIq); MRI; and, in some cases (42 participants), amyloid PET. This population was used to evaluate how CRIq‐based resilience is associated with demographic and clinical variables. CRIq‐based resilience could not be evaluated in the MCI population, as only CN participants had CRIq data available.

All participants signed an informed consent form. The study was approved by the local ethics committee (CCER). The study was conducted according to the Declaration of Helsinki.

### 2.2 Demographics and lifestyle information

Demographic features collected were age, sex, number of years of education, and tobacco consumption (consumer vs. non‐consumer).

### 2.3 Clinical and cognitive measures

Clinical assessment consisted of obtaining a full medical history, including history of cardiovascular disorders, past depression, presence or absence of hypertension, and hypercholesterolemia. Cognitive assessment included a Mini‐Mental State Examination (MMSE) to evaluate global cognition.^20^

### 2.4 Biomarkers

#### 2.4.1 MRI

3T MRI scans were acquired at the Division of Radiology of Geneva University Hospitals using a Magnetom Skyra scanner (Siemens Healthineers) with a 64‐channel head coil. The standardized MRI protocol included a T1‐weighted 3D MPRAGE (magnetization prepared rapid gradient echo) sequence (sagittal acquisition plane, field of view = 240 × 256 × 256 pixels, slice thickness = 1 mm, repetition time/echo time = 1810/2.19 ms, flip angle = 8°, no fat suppression). The T1‐weighted images were used for visually assessing atrophy and for quantitative calculation of gray matter volumes. FreeSurfer software (available for download: http://surfer.nmr.mgh.harvard.edu/) was used for post‐processing with separate segmentation of left‐ and right‐sided regions of interest.^21^

For this study, extracted measures were mean left and right hippocampal volumes (in mm^3^) and, based on a 3D FLAIR (fluid‐attenuated inversion recovery) sequence, volumes of white matter hyperintensities (WMHs; in mm^3^). Mean hippocampal and WMH volumes were normalized to total intracranial volume for each population. In addition, hippocampal volumes were transformed to *z* scores and multiplied by –1 to obtain a neurodegeneration (N) measure.

#### 2.4.2 PET

PET images were acquired using 18F‐flutemetamol, 18F‐florbetapir, and 18F‐flortaucipir at the Nuclear Medicine and Molecular Imaging Division of Geneva University Hospitals using a Biograph mCT or Biograph Vision PET/computed tomography (PET/CT) scanner (Siemens Healthineers), using tracer‐specific protocols. 18F‐florbetapir images were acquired 50 minutes after the intravenous administration of ≈ 200 Mbq of the radiotracer, for 15 minutes. 18‐flutemetamol images were acquired 90 minutes after the injection of ≈ 150 MBq of the radiotracer, for 20 minutes. 18‐flortaucipir images were acquired 75 minutes after the injection of 180 MBq of the radiotracer, for 30 minutes. For all images, we used a 3D ordered subset expectation maximization (OSEM) iterative reconstruction (4 iterations, 12 subsets on the Biograph mCT scanner and 4 iterations, 5 subsets on the Biograph Vision scanner), corrected for randoms, dead time, normalization, scatter, attenuation, and sensitivity.

All PET and MRI images were realigned to the anterior commissure‐posterior commissure line using MATLAB (version 2021a). PET preprocessing was the same for amyloid and tau PET. The preprocessing pipeline had been developed in house at the memory clinic of Geneva University Hospital and published in Dodich et al.^22^ The Centiloid scale was used to obtain a global measure of amyloid load. For global tau load assessment, we used an average of the standardized uptake value ratio (SUVR) extracted from the following regions of interest: parahippocampal gyrus, amygdala, mid‐occipital cortex, and inferior temporal cortex.

#### 2.4.3 Fluid

Blood plasma samples were collected in ethylenediamine tetraacetic acid (EDTA) tubes at the memory clinic of Geneva University Hospitals, kept 2 hours at room temperature before centrifugation (1700 × g for 15 minutes), aliquoted as 500 µL in 1.2 mL polypropylene tubes, and stored at –80°C in the local biobank until the time of genotyping or shipment. For apolipoprotein E (*APOE*) genotyping, realtime TaqMan assay (Applied Biosystems) was performed to test for DNA integrity and quality assessment by electrophoresis. *APOE* genotyping was performed automatically by the same instrument and verified by visual inspection of the generated fluorescence plots. For plasma biomarker quantification, aliquots were shipped on dry ice under protected conditions and analyzed at the Clinical Neurochemistry Laboratory, University of Gothenburg, Sweden. Plasma neurofilament light chain (NfL) and glial fibrillary acidic protein (GFAP) concentrations were measured using commercially available single molecule array (SIMOA) assays on an HD‐X Analyzer according to instructions from the kit manufacturer (Quanterix). Biomarker concentrations were measured by board‐certified laboratory assistants who were blinded to clinical data in two rounds of experiments, using two batches of reagents. For this work, only values extracted from the second and more comprehensive round were used.

### 2.5 Resilience measures

#### 2.5.1 Residual‐based brain and cognitive resilience

- BR: Standardized residuals were extracted from linear models with hippocampal volume as the dependent variable, and A and T as independent variables (hippocampal volume ∼ A*T). The non‐corrected residuals are referred to as BR_non‐cor_
- CR: Standardized residuals were extracted from models using MMSE scores (adjusted for age and education) as the dependent variable, and A, T, and N (hippocampal volume *z* scores *–1) as independent variables (MMSE ∼ A*T*N). The non‐corrected residuals are referred to as CR_n__on‐cor_.

#### 2.5.2 Residual approach correction methods

We compared non‐corrected residuals to residuals corrected using two correction approaches:

- Method 1 (Residual Correction): A secondary regression model was fitted with the original residuals as the dependent variable and the original dependent variable (hippocampal volume or MMSE) as the independent variable. Residuals from this model were considered “corrected residuals” (BR_res‐cor_ and CR_res‐cor_).
- Method 2 (Covariate Correction): The original dependent variable (hippocampal volume or MMSE) was included as a covariate in all subsequent analyses using the residuals as predictors (BR_cov‐cor_ and CR_cov‐cor_).

### 2.6 Statistical analysis

Statistical analyses were conducted using R software version 4.4.1 (https://www.r‐project.org/).

#### 2.6.1 Demographics

To test for differences between males and females (Table S1 in supporting information), Mann–Whitney *U* tests were used for continuous variables, while chi‐squared tests of independence were used for categorical variables.

#### 2.6.2 Association between resilience and factors (aim 1 and 3)

Bivariate linear regression models examined associations between resilience scores and individual variables. For example:

$${\mathrm{BR}}_{\mathrm{non}-\mathrm{cor}}\;∼\;\mathrm{age}$$

For covariate‐corrected resilience scores, multivariable linear regressions were used, with the dependent variable of the regression used to extract residuals added as a covariate, for example:

$${\mathrm{BR}}_{\mathrm{non}-\mathrm{cor}}\;∼\;\mathrm{age}\;+\;\mathrm{Hippocampal}\_\mathrm{volume}$$

#### 2.6.3 Longitudinal analysis (aim 2)

Participants completed a mean (standard deviation [SD]) of 2.24 (1.25) MMSE assessments over a mean (SD) of 3.11 (1.6) years. Linear mixed models were used to assess the influence of resilience on cognitive decline. MMSE change over time was the outcome variable, with the interaction between time and resilience (Time*BR; Time*CR) as the main predictor. Models were adjusted for age and education and included random intercept and slope. For BR_cov‐cor_, the model also included hippocampal volume as a covariate. The covariate correction method was not applied to CR analyses because including the dependent variable (MMSE) as a covariate would have introduced circularity in the model. For visualization purposes, we used a high/low division of resilience (split above/below 0) to show the effects of resilience on cognition over time, but statistical tests were performed using continuous measures of BR and CR.

## 3 RESULTS

### 3.1 Participants

The characteristics of the participants are presented in Table 1. In the MCI population, males and females differed in years of education, prevalence of hypertension, tau load, and carrier prevalence for *APOE* ε4 (Table S1).

### 3.2 Aim 1: association between residual‐based resilience and demographics, clinical features, and biomarkers

**FIGURE 1:**
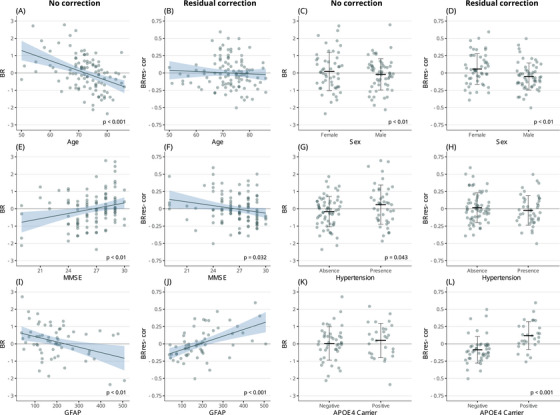
Aim 1: Key associations between non‐corrected versus residual‐corrected measures of brain resilience and demographic, clinical, and biomarker variables in the MCI population. For continuous variables, blue regression lines represent linear fits, with shaded areas indicating 95% confidence intervals, while points display individual values. For categorical variables, jittered points show individual values, and error bars represent means and standard deviations. *APOE*, apolipoprotein E; BR, brain resilience; GFAP, glial fibrillary acidic protein; MCI, mild cognitive impairment; MMSE, Mini‐Mental State Examination.

**FIGURE 2:**
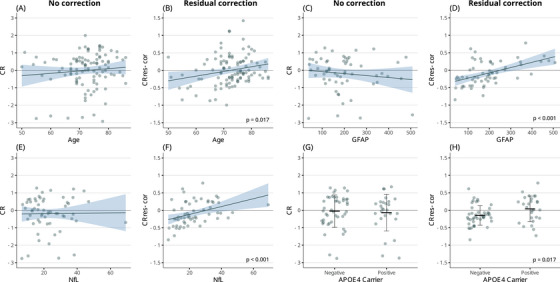
Aim 1: Key associations between non‐corrected versus residual‐corrected measures of cognitive resilience and demographic, clinical, and biomarker variables in the MCI population. For continuous variables, blue regression lines represent linear fits, with shaded areas indicating 95% confidence intervals, while points display individual values. For categorical variables, jittered points display individual values, and error bars represent means and standard deviations. *APOE*, apolipoprotein E; CR, cognitive resilience; GFAP, glial fibrillary acidic protein; MCI, mild cognitive impairment; MMSE, Mini‐Mental State Examination; NfL, neurofilament light chain.

We first examined the relationship between BR and CR and various variables in the MCI population (Figures 1, 2, Tables 2, 3). We found that corrected and non‐corrected residual approaches yielded markedly different associations.

**TABLE 2:** AIM 1: Associations between non‐corrected vs. corrected measures of brain resilience, demographic and clinical characteristics, and biomarkers in the MCI population.

	No correction		Residual Correction		Covariate Correction	
Variable	Standardized β	*p*‐value	Standardized β	*p*‐value	Standardized β	*p*‐value
Age, years	−0.43	**<0.001**	−0.06	0.54	−0.02	0.501
Education, years	−0.09	0.325	0.09	0.361	0.02	0.359
Gender, female	−0.08	0.401	−0.25	**<0.01**	−0.05	**<0.01**
Smoker	<0.01	0.97	−0.17	0.059	−0.04	0.06
Hypertension	0.20	0.043	−0.09	0.361	−0.02	0.351
Depression	−0.05	0.588	−0.15	0.11	−0.03	0.111
Cardiovascular disease	0.04	0.731	−0.18	0.071	−0.04	0.072
Hypercholesterolemia	0.06	0.535	−0.06	0.544	−0.01	0.545
MMSE score	0.25	<0.01	−0.21	**0.032**	−0.05	**0.024**
WMH, mm^3^	−0.18	0.071	−0.16	0.086	−0.04	0.071
GFAP plasma levels	−0.33	<0.01	0.50	**<0.001**	0.13	**<0.001**
NfL plasma levels	−0.33	<0.01	0.21	0.105	0.05	0.091
*APOE* ε4 carrier	0.09	0.451	0.47	**<0.001**	0.10	**<0.001**

*Note*: Values represent standardized beta coefficients (Standardized β) and *p*‐values from bivariate linear models. *N* = 106. Missing values: depression, 1; MMSE score, 2; WMH, 5; GFAP plasma level, 46; NfL plasma level, 46; *APOE* ε4 carriership, 33.

Abbreviations: *APOE*, apolipoprotein E; GFAP, glial fibrillary acidic protein; NfL, neurofilament light chain; WMH, white matter hyperintensities.

**TABLE 3:** AIM 1: Associations between non‐corrected vs. corrected measures of cognitive resilience and demographic, clinical, and biomarkers variables in the MCI population.

	No correction		Residual Correction		Covariate Correction	
Variable	Standardized β	*p*‐value	Standardized β	*p*‐value	Standardized β	*p*‐value
Age, years	0.1	0.322	0.23	**0.017**	0.1	**0.018**
Education, years	0.04	0.675	0.09	0.381	0.04	0.383
Gender, female	0.09	0.353	−0.08	0.402	−0.04	0.4
Smoker	0.02	0.816	−0.1	0.321	−0.1	0.611
Hypertension	0.12	0.239	−0.12	0.206	−0.05	0.2
Depression	0.05	0.596	<0.01	0.948	> −0.01	0.948
Cardiovascular disease	0.11	0.299	−0.15	0.141	−0.07	0.136
Hypercholesterolemia	−0.06	0.523	−0.11	0.28	−0.05	0.282
WMH, mm^3^	−0.13	0.164	−0.09	0.347	−0.04	0.329
GFAP plasma levels	−0.12	0.329	0.39	**<0.001**	0.18	**<0.001**
NfL plasma levels	0.01	0.919	0.3	**<0.01**	0.13	**<0.01**
*APOE* ε4 carrier	−0.04	0.725	0.22	**0.018**	0.1	**0.015**

*Note*: Values represent standardized beta coefficients (Standardized β) and *p*‐values from linear models. *N* = 104. Missing values: Depression, 1; WMH, 4; GFAP plasma levels, 45; NfL plasma levels, 45; *APOE* ε4 carriership, 33.

Abbreviations: *APOE*, apolipoprotein E; GFAP, glial fibrillary acidic protein; NfL, neurofilament light chain; WMH, white matter hyperintensities.

#### 3.2.1 Demographics

Age was negatively associated with non‐corrected BR (BR_non‐cor_: stβ = –0.43, *p* value < 0.001); this association disappeared when BR residuals were corrected using either correction method (Figure 1A,B). The opposite pattern was observed for CR. While age showed no association with CR_non‐cor_, it was positively associated with both corrected CR measures (CR_res‐cor_: stβ = 0.23, *p* value = 0.017; CR_cov‐cor_: stβ = 0.1, *p* value = 0.018, Figure 2A,B).

Female sex presented no significant association with non‐corrected BR, while it was associated with BR residuals corrected using either correction method (BR_res‐cor_: stβ = 0.25, *p* value < 0.01; BR_cov‐cor_: stβ = 0.05, *p* value < 0.01, Figure 1C,D).

A trend for a negative association was observed between smoking and corrected BR measures, but not BR_non‐cor_ (BR_res‐cor_: stβ = –0.17, *p*‐value = 0.059; BR_cov‐cor_: stβ = –0.04, *p*‐value = 0.06).

#### 3.2.2 Clinical features

MMSE score was significantly associated with all BR measures, but the direction of the association was reversed between non‐corrected and corrected BR (BR_non‐cor_: stβ = 0.25, *p*‐value < 0.01; BR_res‐cor_: stβ = –0.21, *p*‐value = 0.032; BR_cov‐cor_: stβ = –0.05, *p*‐value = 0.024, Figure 1E,F).

A positive association was found between hypertension and BR_non‐cor_ (stβ = 0.2, *p*‐value = 0.043), while this association was not present with either of the corrected BR measures (Figure 1G,H).

A trend for a negative association was observed between history of cardiovascular disease and corrected BR measures, but not BR_non‐cor_ (BR_res‐cor_: stβ = –0.18, *p*‐value = 0.071; BR_cov‐cor_: stβ = –0.04, *p*‐value = 0.072).

#### 3.2.3 Biomarkers

A trend for a negative association was observed between WMH volume and all BR measures (BR_non‐cor_: stβ = –0.18, *p*‐value = 0.071; BR_res‐cor_: stβ = –0.16, *p*‐value = 0.086; BR_cov‐cor_: stβ = –0.04, *p*‐value = 0.071).

GFAP plasma levels were significantly associated with all BR measures, but the direction of the association was reversed between non‐corrected and corrected BR measures (BR_non‐cor_: stβ = –0.33, *p*‐value < 0.01; BR_res‐cor_: stβ = 0.5, *p*‐value < 0.001; BR_cov‐cor_: stβ = 0.13, *p*‐value < 0.001, Figure 1I,J). CR_non‐cor_ did not show an association with GFAP plasma levels, while both corrected CR measures were positively associated with this variable (CR_res‐cor_: stβ = 0.39, *p*‐value = < 0.001; CR_cov‐cor_: stβ = 0.18, *p*‐value < 0.001, Figure 2C,D).

NfL plasma levels were negatively associated with BR_non‐cor_ (stβ = –0.33, *p*‐value < 0.01), but not with corrected BR, although a trend was observed for a positive association with BR_cov‐cor_ (BR_cov‐cor_: stβ = 0.05, *p*‐value = 0.091). CR_non‐cor_ did not show an association with NfL plasma levels, while both corrected CR measures were positively associated with this variable (CR_res‐cor_: stβ = 0.3, *p*‐value < 0.01; CR_cov‐cor_: stβ = 0.13, *p*‐value < 0.01, Figure 2E,F).

*APOE* ε4 carrier prevalence was not associated with BR_non‐cor_ but was positively associated with both corrected BR measures (BR_res‐cor_: stβ = 0.47, *p*‐value < 0.001; BR_cov‐cor_: stβ = 0.1, *p*‐value < 0.001, Figure 1K,L). The same pattern was observed for CR, with CR_non‐cor_ presenting no association with *APOE* ε4 carrier prevalence, while both corrected CR measures showed a positive association with this variable (CR_res‐cor_: stβ = 0.22, *p*‐value = 0.018; CR_cov‐cor_: stβ = 0.1, *p*‐value = 0.015, Figure 2G,H).

#### 3.2.4 CRIq‐based CR

To further explore the factors influencing resilience, we investigated associations with an alternate CR measure, the CRIq. Our goal was to identify any common factors associated with both residual‐based and CRIq‐based measures of cognitive resilience. As CRIq data were unavailable for our MCI population, we examined these associations in a separate sample of CN subjects (*n* = 124). In this population, older age was associated with higher CRIq scores (stβ = 0.29, *p*‐value < 0.01; Table S2 in supporting information).

### 3.3 Aim 2: influence of corrected versus non‐corrected residuals on cognitive decline

**FIGURE 3:**
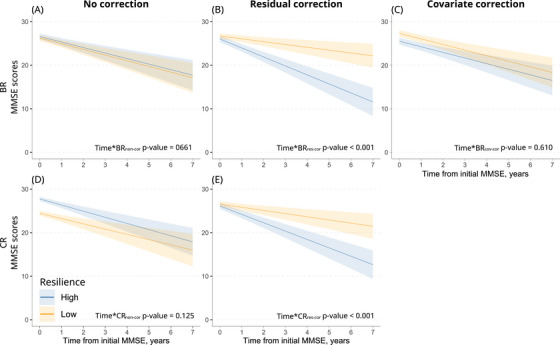
Aim 2: Impact of non‐corrected and corrected residual‐based resilience on longitudinal MMSE scores in the MCI population. Predicted MMSE scores over time for high and low BR_non‐cor_, BR_res‐cor,_ and BR_cov‐cor_ (top row) and for high and low CR_non‐cor_ and CR_res‐cor_ (bottom row) groups. Models are adjusted for age and education. High and low resilience groups were used for visualization purposes, but statistical tests were performed using continuous resilience variables. Blue and orange lines represent high and low resilience, respectively, with shaded areas indicating 95% confidence intervals. BR, brain resilience; CR, cognitive resilience; MCI, mild cognitive impairment; MMSE, Mini‐Mental State Examination.

Next, we looked at the ways residual‐based BR and CR influenced the trajectory of cognitive performance over time using linear mixed effect models in the MCI population. We again observed marked differences between corrected and non‐corrected residuals (Figure 3, Tables S3‐S4 in supporting information). While neither BR_non‐cor_ nor CR_non‐cor_ showed a significant effect on cognition over time (Figure 3A,D), both BR_res‐cor_ and CR_res‐cor_ presented a strong negative influence on cognitive trajectories (Time* BR_res‐cor_: β = –3.89, *p*‐value < 0.001; Time*CR_res‐cor_: β = –1.53, *p*‐value < 0.001, Figure 3B,E). Using the covariate correction (only possible for BR, see the Methods section) did not show any significant interaction of Time* BR_cov‐cor_ (Figure 3C).

## 4 DISCUSSION

This study aimed to evaluate the validity of the residual approach by comparing corrected and non‐corrected residual approaches in the assessment of resilience in a memory clinic population. Our findings revealed important differences between the alternative methods. First, they yielded markedly different, and sometimes opposite, associations with demographic and clinical factors. The choice of residual correction also significantly altered the observed relationship between resilience and cognitive decline. While non‐corrected resilience scores showed little influence on cognitive trajectories, residual‐corrected resilience was strongly predictive of long‐term cognitive performance, with higher resilience being associated with worse cognition.

Ossenkoppele et al.^9^ also used the residual approach to identify factors associated with BR and CR to tau pathology. They found that younger age, female sex, and lower WMH volumes were associated with greater BR, and that younger age, higher education, lower WMH, and greater cortical thickness were associated with greater CR in bivariate models. Importantly, they used only a non‐corrected residual approach. Our results partially align with that group's for non‐corrected residuals, as we found an association between greater BR_non‐cor_ and younger age, and a trend for association with lower WMH volumes (Tables S5‐S6 in supporting information). However, upon correcting residuals, we observed considerably different, and sometimes opposite, associations.

**FIGURE 4:**
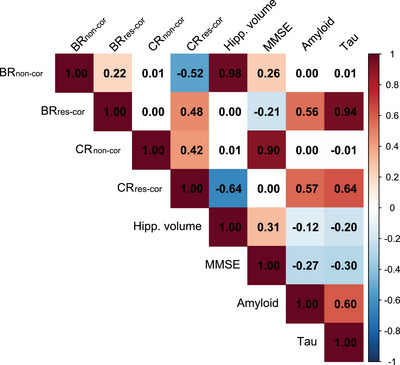
Correlations between variables used for residual calculation and resulting residuals. Values represent Pearson correlation coefficients. BR, brain resilience; CR, cognitive resilience; MMSE, Mini‐Mental State Examination.

These discrepancies stem from the inherent correlation between residuals and the variables of the regression used to extract them, a correlation that strengthens when the relationship between these variables is weak (Figure 4). Because of this association, factors that correlate with the dependent variable appear associated with the non‐corrected resilience score, even if they do not represent true resilience mechanisms. As correcting residuals shifts their association from the dependent variable (brain or cognitive measure) to the independent variable (neuropathology), factors more closely aligned with neuropathology (e.g., prevalence of *APOE* ε4 carriers) show an association with corrected, but not non‐corrected, residuals. Factors associated with both the dependent and independent variables (e.g., MMSE or GFAP for BR) show associations with both corrected and non‐corrected residuals, with opposite directionality.

In a separate CN population, older age was associated with higher CRIq‐based CR scores. However, caution is warranted in interpreting this, as the CRIq assesses factors (education, occupation, leisure, social activity) that naturally accumulate with age, leading to older individuals usually exhibiting higher CRIq scores.^6^ Moreover, while the factors used to calculate the CRIq contribute to overall cognitive health, their direct link to resilience remains unclear. For example, higher education might correlate with better access to health care or healthier lifestyles, influencing brain health without necessarily promoting resilience. Thus, although the CRIq may provide valuable insights, its ability to isolate specific resilience factors warrants further investigation.

Studying the longitudinal impact of resilience on cognition, Ossenkoppele et al.^9^ found a significant correlation between BR and CR, and a significant interaction between BR and CR on cognitive decline. In contrast, our non‐corrected measures were uncorrelated and did not predict decline. However, correcting residuals profoundly influenced the observed relationship between resilience and cognitive trajectory. Both BR_res‐cor_ and CR_res‐cor_ showed a strong negative effect on cognition over time. This occurs for the same reasons as described above: correcting residuals shifts their association from the dependent variable to the measure of neuropathology. As a high level of pathology is probably a better predictor of cognitive decline than either hippocampal volume or initial MMSE score (i.e., the dependent variables), BR_res‐cor_ and CR_res‐cor_ seem to have a greater influence on cognitive trajectories than BR_non‐cor_ and CR_non‐cor_. The covariate correction for BR showed mixed results, not predicting decline but exhibiting a negative association with baseline cognition. The results underline how the choice of a residual correction approach can lead to profoundly different interpretations of the influence of resilience on disease progression.

### 4.1 Limitations

This study has limitations that should be acknowledged. First, our sample was limited to a memory clinic population with MCI. While this allowed for a focused analysis of the residual approach in a clinically relevant population, it limits the generalizability of our findings to other populations, such as CN individuals or those with dementia. Future studies should examine the residual approach in more diverse samples to assess the consistency of our findings across different stages of cognitive decline.

Second, our study relied on the MMSE as the primary measure of cognitive function. The MMSE is subject to ceiling effects and lacks the precision of dedicated episodic memory tests. However, it provides a global cognition assessment and is commonly used in clinical practice, research studies, and clinical trials. Furthermore, our primary goal was to examine the limitations of the residual approach itself, which are evident regardless of the specific cognitive measure used.

### 4.2 Conclusion

Our findings demonstrate that the residual approach, while seemingly straightforward, has significant limitations. The choice of correction method and the inherent correlations between residuals and the variables used to extract them can drastically influence the results and lead to misinterpretations. This calls into question the ability of this approach to truly capture resilience. Researchers and clinicians should be aware of these limitations and consider alternative approaches. At the minimum, the results of the residual approach should be interpreted with caution.

It is tempting for researchers and clinicians to rely on quick, cross‐sectional methods to obtain individual resilience scores that can be used to identify modifiable risk factors. While these methods can offer valuable tools for initial exploration, one must recognize that factors influencing general brain and cognitive health might overlap with, but are not necessarily synonymous with, true resilience mechanisms. To truly comprehend the complex nature of resilience, we likely need to shift toward more sophisticated approaches. These could include longitudinal studies that track brain and cognitive changes over long periods, considering a wide range of life events and influencing factors. Additionally, more complex statistical models, such as structural equation modeling, might offer a more nuanced and accurate assessment of resilience.

By acknowledging the inherent biases of each method and actively exploring alternative approaches, the field can move toward a more comprehensive and nuanced understanding of resilience in AD. We urge researchers to critically evaluate the limitations of current methods and prioritize the development of more robust and clinically meaningful approaches for assessing resilience.

## CONFLICTS OF INTEREST STATEMENT

HZ has served on scientific advisory boards and/or as a consultant for Abbvie, Acumen, Alector, Alzinova, ALZpath, Amylyx, Annexon, Apellis, Artery Therapeutics, AZTherapies, Cognito Therapeutics, CogRx, Denali, Eisai, LabCorp, Merry Life, Nervgen, Novo Nordisk, Optoceutics, Passage Bio, Pinteon Therapeutics, Prothena, Quanterix, Red Abbey Labs, reMYND, Roche, Samumed, Siemens Healthineers, Triplet Therapeutics, and Wave; has given lectures sponsored by Alzecure, BioArctic, Biogen, Cellectricon, Fujirebio, Lilly, Novo Nordisk, Roche, and WebMD; and is a co‐founder of Brain Biomarker Solutions in Gothenburg AB (BBS), which is a part of the GU Ventures Incubator Program (outside submitted work). KB has served as a consultant and on advisory boards for Acumen, ALZPath, AriBio, BioArctic, Biogen, Eisai, Lilly, Moleac Pte. Ltd, Novartis, Ono Pharma, Prothena, Roche Diagnostics, and Siemens Healthineers; has served on data monitoring committees for Julius Clinical and Novartis; has given lectures, produced educational materials, and participated in educational programs for AC Immune, Biogen, Celdara Medical, Eisai, and Roche Diagnostics; and is a co‐founder of Brain Biomarker Solutions in Gothenburg AB (BBS), which is a part of the GU Ventures Incubator Program, outside the work presented in this paper. NJA has received consulting fees from Athria, ImaginationLand LLC, MapLight Therapeutics, SpeaBio, Neurogen Biomarking, Quanterix, TauRx; has given lectures sponsored by Alamar Biosciences, Biogen, Eli‐Lilly, Quanterix, VJDementia; and has participated on advisory boards for Biogen, TargetALS, and TauRx. VG received research support and speaker fees through her institution from GE Healthcare, Siemens Healthineers, Novo Nordisk, Janssen, and Novartis. GBF has received support, payment, consulting fees, or honoraria through his institution for lectures, presentations, speaker bureaus, manuscript writing, or educations events from: Biogen, Roche, Diadem, Novo Nordisk, GE Healthcare, OM Pharma, and Eisai. The other authors have no conflicts of interest to disclose.

## CONSENT STATEMENT

All participants signed an informed consent form.

## Supporting information



Supporting information

Supporting information
