# Supplementary material for: Resilience in Alzheimer's disease: Impact of operationalization and methodological choices
Source: Alzheimers Dement. 2025 Apr 28;21(4):e70185. doi: 10.1002/alz.70185 (PMC12035551; doi:10.1002/alz.70185)
Supplement: Supplementary file 2 — Supporting information [file ALZ-21-e70185-s001.docx]

# Supplementary Materials

**Supplementary Table 1. Gender differences in MCI and CN populations.**

**Supplementary Table 2. AIM 3: Associations between measures of CRIq-based cognitive resilience and demographic, clinical, and biomarker variables in the CN population.**

**Supplementary Table 3. AIM 2: Linear mixed-effects model results: Impact of non-corrected and corrected BR on longitudinal MMSE scores in the MCI population.**

**Supplementary Table 4. AIM 2: Linear mixed-effects model results: Impact of non-corrected and corrected CR on longitudinal MMSE scores in the MCI population.**

**Supplementary Table 5. Brain resilience and associated factors: Current study vs. Ossenkoppele et al. (2020).**

**Supplementary Table 6. Cognitive resilience and associated factors: Current study vs. Ossenkoppele et al. (2020).**

**Supplementary Table 1. Gender differences in MCI and CN populations.**

|  | **MCI (n = 121)** | | | | **CN (n = 124)** | | |
| --- | --- | --- | --- | --- | --- | --- | --- |
| **Variable** | **Male (66)** | **Female (55)** | **P-value** | | **Male (34)** | **Female (90)** | **P-value** |
| Age, years | 72.8 (7.3) | 71.9 (7.6) | | 0.256 | 62.5 (8.8) | 61.4 (8.7) | 0.616 |
| Education, years | 14.1 (3.8) | 12.7 (3.5) | | **0.03** | 15.3 (2.6) | 15.5 (3.8) | 0.966 |
| Smoker | 6 (9) | 3 (5) | | 0.681 | 2 (6) | 8 (9) | 1 |
| Hypertension | 32 (48) | 16 (29) | | **0.047** | 5 (15) | 9 (10) | 0.46 |
| Depression | 13 (20) | 16 (29) | | 0.345 | 6 (18) | 24 (27) | 0.693 |
| Cardiovascular disease | 13 (20) | 7 (13) | | 0.434 | 5 (15) | 8 (9) | 0.404 |
| Hypercholesterolemia | 26 (39) | 24 (44) | | 0.774 | 7 (21) | 11 (12) | 0.187 |
| MMSE score | 26.8 (2.5) | 26 (2.5) | | 0.072 | 28.6 (1.2) | 28.8 (1) | 0.537 |
| Hippocampal volume, mm^3^ | 7012 (853) | 7103 (1075) | | 0.792 | 7938 (1421) | 8401 (850) | 0.438 |
| Amyloid centiloid | 43.7 (48.6) | 57.1 (49.2) | | 0.161 | 14.3 (20.6) | 2 (12.8) | 0.08 |
| Tau SUVr | 1.3 (0.2) | 1.4 (0.3) | | **< 0.01** | NA | NA | NA |
| WMH, mm3 | 5240.5 (10273) | 4429.9 (6659) | | 0.785 | NA | NA | NA |
| GFAP plasma levels | 173.6 (105.9) | 210.8 (111.3) | | 0.144 | NA | NA | NA |
| NfL plasma levels | 21.3 (9.1) | 24.8 (13.1) | | 0.324 | NA | NA | NA |
| *APOE* ε4 carrier | 11 (17) | 20 (36) | | **0.034** | 11 (32) | 23 (26) | 0.448 |

Values represent means (SD) for continuous variables and n (%) for categorical variables. Mann-Whitney tests were used to assess differences between groups for continuous variables, while Chi-squared tests of independence were used to assess differences between groups for categorical variables.

**Supplementary Table 2. AIM3 : Associations between measures of CRIq-based cognitive resilience and demographic, clinical, and biomarker variables in the CN population.**

| **Variable** | **Standardized β** | **P-value** |
| --- | --- | --- |
| Age, years | 0.29 | **< 0.01** |
| Gender, female | 0.07 | 0.45 |
| Smoker | -0.05 | 0.576 |
| Hypertension | 0.17 | 0.099 |
| Depression | -0.11 | 0.31 |
| Cardiovascular disease | -0.02 | 0.858 |
| Hypercholesterolemia | 0.14 | 0.184 |
| MMSE score | 0.02 | 0.835 |
| Hippocampal vol., mm^3^ | 0.18 | 0.256 |
| Amyloid centiloid | -0.02 | 0.911 |
| *APOE* ε4 carrier | 0.04 | 0.639 |

Values represent standardized beta coefficients (standardized β) and p-values from bivariate linear models. N = 124. Missing values: smoker, 20; hypertension, 21; depression, 21; cardiovascular disease, 25; Hypercholesterolemia, 20; MMSE score, 3; hippocampal vol., 69; amyloid centiloid, 85; *APOE* ε4 carriership, 9.

**Supplementary Table 3. AIM 2: Linear mixed-effects model results: Impact of non-corrected and corrected brain resilience on longitudinal MMSE scores in the MCI population.**

|  | **No correction** | | **Residual Correction** | | **Covariate Correction** | |
| --- | --- | --- | --- | --- | --- | --- |
| **Variable** | **β (SE)** | **P-value** | **β (SE)** | **P-value** | **β (SE)** | **P-value** |
| Age, years | 0.04 (0.04) | 0.281 | 0.02 (0.04) | 0.695 | 0.05 (0.04) | 0.216 |
| Education, years | 0.03 (0.07) | 0.619 | 0.04 (0.07) | 0.542 | 0.06 (0.07) | 0.379 |
| Hippocampal volume, mm^3^ | NA | NA | NA | NA | < 0.01 (<0.01) | **< 0.001** |
| Time | -1.22 (0.18) | **< 0.001** | -1.13 (0.14) | **< 0.001** | -1.19 (0.18) | **< 0.001** |
| BR | 0.69 (0.32) | **< 0.01** | -1.63 (1.23) | 0.188 | -2.36 (1.18) | **0.045** |
| Time*BR | 0.09 (0.2) | 0.661 | -3.79 (0.68) | **< 0.001** | 0.11 (0.2) | 0.610 |

Values represent beta coefficients (SE).

**Supplementary Table 4. AIM 2: Linear mixed-effects model results: Impact of non-corrected and corrected cognitive resilience on longitudinal MMSE scores in the MCI population.**

|  | **No correction** | | **Residual Correction** | |
| --- | --- | --- | --- | --- |
| **Variable** | **β (SE)** | **P-value** | **β (SE)** | **P-value** |
| Age, years | 0.01 (0.02) | 0.381 | 0.01 (0.07) | 0.825 |
| Education, years | 0.04 (0.04) | 0.277 | 0.02 (0.07) | 0.838 |
| Time | -1.28 (0.18) | **< 0.001** | -1.24 (0.16) | **< 0.001** |
| CR | 2.15 (0.15) | **< 0.001** | 0.67 (0.66) | 0.307 |
| Time*CR | -0.29 (0.19) | 0.125 | -1.45 (0.37) | **< 0.001** |

Values represent beta coefficients (SE).

**Supplementary Table 5. Brain resilience and associated factors: Current study vs. Ossenkoppele et al. (2020).**

|  | **No correction** |  | **Residual Correction** | | **Covariate Correction** | | **Ossenkoppele et al.** | |
| --- | --- | --- | --- | --- | --- | --- | --- | --- |
| **Variable** | **Standardized β** | **P-value** | **Standardized β** | **P-value** | **Standardized β** | **P-value** | **Standardized β** | **P-value** |
| Age, years | -0.43 | **< 0.001** | -0.06 | 0.54 | -0.02 | 0.501 | -0.3 | **< 0.001** |
| Education, years | -0.09 | 0.325 | 0.09 | 0.361 | 0.02 | 0.359 | -0.05 | 0.43 |
| Sex, female | 0.08 | 0.401 | 0.25 | **< 0.01** | 0.05 | **< 0.01** | 0.19 | **0.003** |
| WMH, mm^3^ | -0.18 | 0.071 | -0.16 | 0.086 | -0.04 | 0.071 | -0.28 | **< 0.001** |
| *APOE* ε4 carrier | 0.09 | 0.451 | 0.47 | **< 0.001** | 0.1 | **< 0.001** | 0.04 | 0.53 |

Values represent standardized beta coefficients (standardized β) and p-values from bivariate linear models.

**Supplementary Table 6. Cognitive resilience and associated factors: Current study vs. Ossenkoppele et al. (2020).**

|  | **No correction** | | **Residual Correction** | | **Covariate Correction** | | **Ossenkoppele et al.** | |
| --- | --- | --- | --- | --- | --- | --- | --- | --- |
| **Variable** | **Standardized β** | **P-value** | **Standardized β** | **P-value** | **Standardized β** | **P-value** | **Standardized β** | **P-value** |
| Age, years | 0.1 | 0.322 | 0.23 | **0.017** | 0.1 | **0.018** | -0.17 | **0.008** |
| Education, years | 0.04 | 0.675 | 0.09 | 0.381 | 0.04 | 0.383 | 0.26 | **< 0.001** |
| Sex, female | -0.09 | 0.353 | 0.09 | 0.347 | 0.04 | 0.329 | -0.12 | 0.85 |
| WMH, mm^3^ | -0.13 | 0.164 | 0.3 | **< 0.01** | 0.13 | **< 0.01** | -0.24 | **< 0.001** |
| *APOE* ε4 carrier | -0.04 | 0.725 | 0.22 | **0.018** | 0.1 | **0.015** | 0.24 | **< 0.001** |

Values represent standardized beta coefficients (standardized β) and p-values from bivariate linear models.
